# Emerging ImmunoPET probes for precision cancer immunotherapy: molecular targets and translational applications

**DOI:** 10.3389/fonc.2026.1893952

**Published:** 2026-07-22

**Authors:** Ting Liu, Jiamao Lin, Yang Li, Yong Wang

**Affiliations:** 1 Department of Clinical Nutrition, Ordos Central Hospital, Ordos, China; 2 Department of Traditional Chinese and Western Medicine, Shandong Cancer Hospital and Institute, Shandong First Medical University and Shandong Academy of Medical Sciences, Jinan, China; 3 Shandong Provincial Key Laboratory of Precision Oncology, Shandong Cancer Hospital and Institute, Shandong First Medical University and Shandong Academy of Medical Sciences, Jinan, China; 4 Department of Radiation Oncology, Shandong Cancer Hospital and Institute, Shandong First Medical University and Shandong Academy of Medical Sciences, Jinan, China

**Keywords:** bioorthogonal chemistry, ImmunoPET, PD-1/PD-L1, precision immunotherapy, radiolabeled antibody, theranostics, tumor microenvironment

## Abstract

Immune checkpoint inhibitors have transformed oncology, yet durable responses remain unevenly distributed, and existing stratification tools—single-site biopsy, PD-L1 immunohistochemistry, and [¹^8^F]FDG-PET—fail to capture the spatial heterogeneity and dynamic evolution of the tumor immune microenvironment. ImmunoPET addresses these limitations by pairing antibody-based molecular recognition with whole-body quantitative PET, enabling non-invasive mapping of checkpoint expression across the entire disease burden. This review examines the biological rationale and translational status of targets spanning inhibitory axes (PD-1/PD-L1, CTLA-4), next-generation co-inhibitory receptors (LAG-3, TIM-3, TIGIT, VISTA), and co-stimulatory targets (ICOS, 4-1BB, B7-H3), alongside probe engineering principles including scaffold selection, radionuclide pairing, and bioorthogonal pretargeting. Clinical evidence across thoracic, genitourinary, hematological, and neuro-oncological contexts demonstrates that whole-body PET metrics outperform concurrent IHC in predicting treatment outcomes. Theranostic extensions and radiomics applications are further discussed. Standardizing quantitative thresholds and harmonizing acquisition protocols remain the critical steps toward regulatory qualification of ImmunoPET as a companion diagnostic.

## 1 Introduction

Immune checkpoint inhibitors (ICIs) have substantially changed the treatment of advanced malignancies. Since the approval of ipilimumab in 2011, agents targeting the PD-1/PD-L1 and CTLA-4 axes have demonstrated durable responses across more than twenty tumor types, and chimeric antigen receptor T-cell (CAR-T) therapy has extended this reach to hematological malignancies (1–5). Clinical benefit, however, is unevenly distributed. Objective response rates vary substantially across tumor types and individual patients, roughly half of treated patients develop immune-related adverse events (irAEs), and a subset experience hyperprogression following ICI initiation (6–9). These outcomes cannot be reliably predicted with existing tools.

Patient stratification currently depends on immunohistochemical (IHC) assessment of PD-L1 expression and CD8^+^ T-cell density in single-site biopsies. This approach captures only a fraction of the tumor burden and fails to represent immunological heterogeneity across metastatic lesions (10–12). The tumor microenvironment (TME) also evolves under treatment pressure: antigen loss, clonal selection, and T-cell exhaustion occur on timescales that a static biopsy cannot resolve (13–15). The clinical record reflects this directly. IHC-negative patients sometimes achieve durable remissions; IHC-positive patients may not respond. Conventional imaging adds little specificity. CT and MRI measure size changes that lag behind the underlying biology and cannot distinguish immune infiltration from true progression (16, 17). [^18^F]FDG-PET provides metabolic information, but activated T cells and proliferating tumor cells share glycolytic upregulation, producing signal that cannot reliably identify the nature of an immune response (18–20).

ImmunoPET addresses these limitations directly by pairing antibody-based molecular recognition with whole-body PET quantification (21). Intact IgG constructs labeled with ^89^Zr (t_1/2_ = 78.4 h) or ^64^Cu (t_1/2_ = 12.7 h) accommodate slow antibody pharmacokinetics, while smaller scaffolds, including single-domain antibodies and affibodies, are matched to ^68^Ga (t_1/2_ = 68 min) or ^18^F (t_1/2_ = 110 min) for same-day imaging (22, 23). A single acquisition maps target expression across the entire disease burden; repeat imaging tracks immune dynamics longitudinally without biopsy. Clinical studies have already demonstrated that whole-body ImmunoPET metrics predict treatment outcomes more accurately than concurrent IHC (24, 25). This review examines the biological rationale, probe design principles, current tracer landscape, clinical applications, and outstanding challenges of ImmunoPET in the context of precision cancer immunotherapy. To provide a clear methodological overview for precision clinical stratification, the core biological principles, molecular targets, diagnostic advantages, and limitations of ImmunoPET, ^18^F-FDG PET/CT, and immunohistochemistry (IHC) are systematically contrasted in **Table 1**.

**Table 1:** Comparative matrix of ImmunoPET, ¹^8^F-FDG PET/CT, and immunohistochemistry (IHC) in precision cancer immunotherapy.

Aspect	ImmunoPET	¹^8^F-FDG PET/CT	Immunohistochemistry (IHC)
Biological principle	Target-specific *in vivo* imaging of immune checkpoints, immune cells, or stromal markers using radiolabeled antibodies or engineered scaffolds.	Imaging of glucose metabolism reflecting cellular glycolytic activity in tumor and inflammatory cells.	Ex vivo antigen–antibody detection in formalin-fixed tissue sections.
What it measures	Spatial distribution and expression level of immune-related targets across the whole tumor burden.	Whole-body metabolic activity based on glucose uptake and phosphorylation via GLUT1/GLUT3 pathways.	Local protein expression within a single biopsy specimen.
Molecular targets	PD-1/PD-L1, CTLA-4, LAG-3, TIM-3, TIGIT, VISTA, ICOS, 4-1BB, B7-H3 (CD276).	Non-specific metabolic uptake mediated by glucose transporters.	PD-L1, PD-1, CD8, FOXP3, and panel-dependent biomarkers.
Spatial coverage	Whole-body, lesion-level assessment including primary and metastatic sites.	Whole-body metabolic imaging of all detectable lesions.	Single or limited biopsy site; limited representation of spatial heterogeneity.
Temporal capability	Enables longitudinal, repeatable imaging of immune dynamics during therapy.	Repeated metabolic imaging; reflects time-dependent changes in tumor burden.	Static snapshot; longitudinal assessment requires repeat invasive biopsies.
Specificity	High molecular specificity for predefined immune targets.	Low biological specificity; cannot distinguish tumor progression from inflammation or immune activation.	High analytical specificity but limited by sampling representativeness.
Quantification	Quantitative metrics including SUV, TBR, kinetic modeling (e.g., Patlak *K*_i_), and radiomics.	Semi-quantitative metrics including SUVmax, MTV, and TLG.	Semi-quantitative scoring systems (H-score, CPS, TPS).
Sensitivity to heterogeneity	High sensitivity to inter- and intra-lesional immune heterogeneity.	Moderate sensitivity to metabolic heterogeneity.	Low sensitivity; strongly affected by sampling bias.
Clinical applications	Immunotherapy stratification, response prediction, immune engagement monitoring, and emerging theranostic applications.	Tumor staging, baseline burden assessment, and metabolic response evaluation (PERCIST).	Companion diagnostic testing for treatment selection and biomarker validation.
Advantages	Whole-body immune profiling; target specificity; non-invasive; repeatable longitudinal assessment.	Widely available; standardized protocols; cost-effective; rapid imaging workflow.	High molecular specificity; established clinical pathology standard.
Limitations	Limited tracer availability; complex GMP production; evolving regulatory standardization and validation frameworks.	Non-specific uptake in inflammation or immune activation may confound interpretation.	Invasive; spatial sampling bias; limited suitability for longitudinal monitoring.
Role in precision oncology	Emerging imaging biomarker for immunotherapy guidance and systemic immune profiling.	Standard tool for staging and metabolic response assessment.	Established diagnostic reference for baseline biomarker evaluation, but limited systemic representativeness.
Overall clinical value	Provides systemic, target-specific visualization of tumor–immune interactions.	Provides robust global metabolic assessment of disease burden.	Provides gold-standard tissue-level validation with limited spatial and temporal coverage.

## 2 The checkpoint target landscape: from established axes to emerging molecules

**Figure 1:**
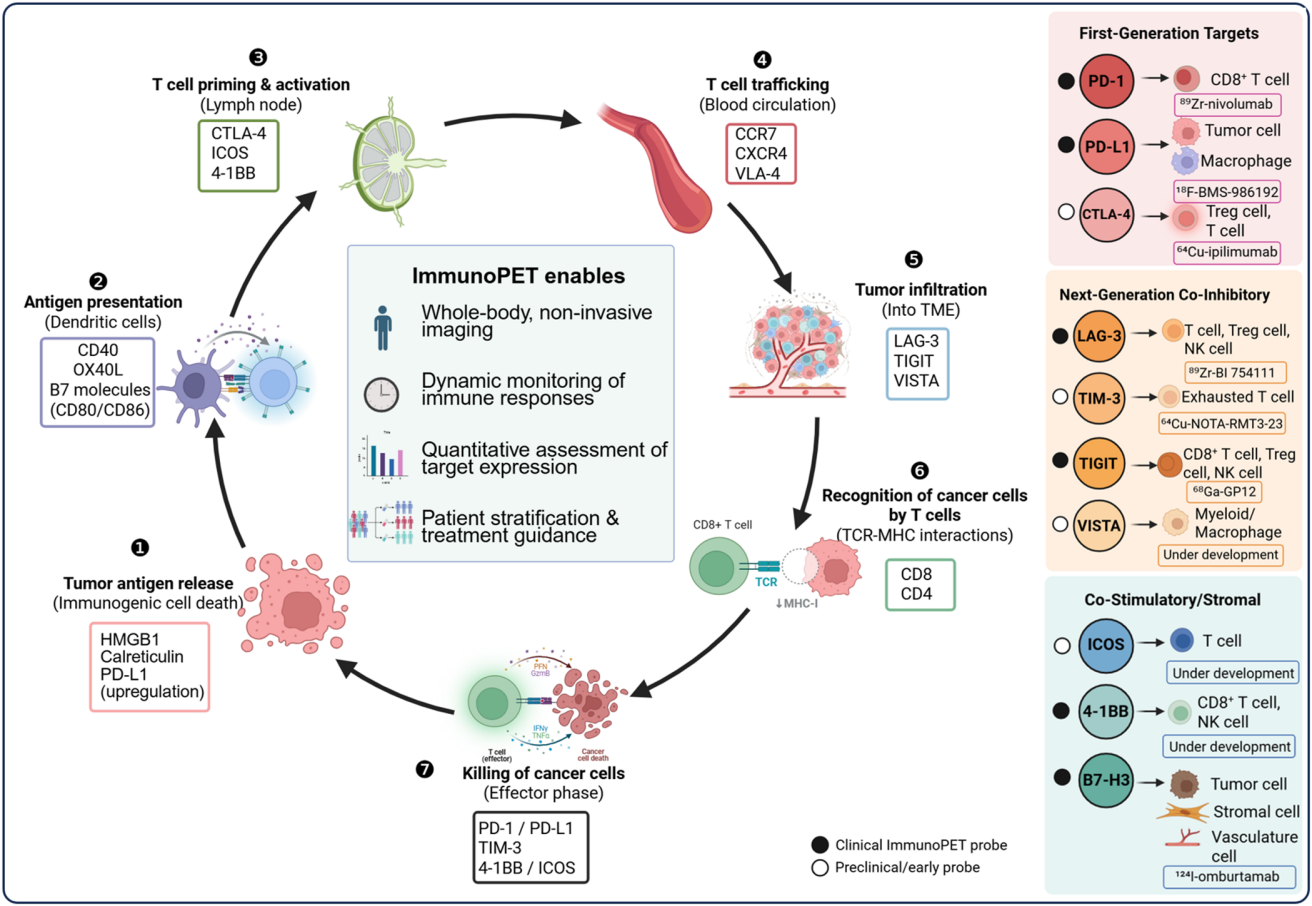
Schematic illustration of the cancer-immune cycle integrated with first-generation, next-generation co-inhibitory, and co-stimulatory ImmunoPET biomarkers. Solid circles (○) indicate tracers in clinical validation stages (Phase I/II), whereas open circles (○) represent preclinical or early exploratory probes.

Immune checkpoints span a functionally heterogeneous network of inhibitory and co-stimulatory receptors (26). Their expression patterns differ across cell types, tumor histologies, and treatment contexts (27). This diversity demands an organized framework for ImmunoPET target selection—one grounded in receptor biology, therapeutic precedent, and the specific clinical question each imaging probe is designed to answer (28). To systematically visualize these interactions, the targets discussed in this section are integrated within the multi-step Cancer-Immune Cycle in **Figure 1**, where they are organized by functional class and annotated with representative clinical or preclinical probes alongside their current translational stage. Furthermore, a comprehensive, multi-dimensional taxonomy of these first-generation, next-generation co-inhibitory, and co-stimulatory targets—delineating their premier representative benchmark tracers, host scaffold characteristics, clinical evaluation phases, and core imaging insights—is systematically curated in **Table 2**.

**Table 2:** ImmunoPET target portfolio: checkpoint, co-stimulatory, and stromal targets.

Target category	Representative tracers	Scaffold type	Translational stage	Key insights & imaging findings	Reference
PD-1 (First-Generation)	^89^Zr-pembrolizumab	Intact IgG (~150 kDa)	Phase I/II Clinical Advanced NSCLC, Metastatic Melanoma	• Extended Kinetics: Requires a 7-day clearance window; optimal cold mass dose established at 5 mg. • Survival Predictor: Baseline tumor uptake significantly correlates with objective response, PFS, and OS. • Lymphoid Sink: Demonstrates prominent physiologic tracer retention in the spleen and normal lymphoid tissues.	(25)
PD-L1 (First-Generation)	^18^F-BMS-986192	Adnectin (∼12 kDa)	Phase I/II Clinical Advanced NSCLC	• Rapid Optimization: Short scaffold enables rapid blood clearance and same-day target quantification at 60 min. • Validated Modeling: Confirmed to fit a single-tissue reversible compartment model via arterial blood sampling. • Surrogate Reliability: Standardized uptake value (SUV) tightly correlates with true distribution volume (V_T_, *R*^2^ > 0.9).	(36)
CTLA-4 (First-Generation)	^64^Cu-DOTA-ipilimumab	Intact IgG (~150 kDa)	Preclinical NSCLC murine models	• Expression Mapping: Non-invasively characterizes intrinsic CTLA-4 levels on solid tumors, with tracer accumulation scaling with baseline cellular expression. • Persistent Kinetics: Exhibits enhanced and continuous tracer accumulation in target tissues during longitudinal PET up to 48 h post-injection. • Target Specificity: *In vivo* binding specificity fully validated via cold antibody blocking, significantly reducing tumor tracer retention.	(42)
LAG-3 (Next-Gen Co-inhibitory)	^89^Zr-BI-754111	Intact IgG (~150 kDa)	Phase I Clinical Advanced NSCLC & HNSCC	• Clearance & Imaging Window: Favorable longitudinal kinetics establish the optimal whole-body PET target delineation at 96 h to 144 h post-injection. • Immunological Correlation: Intratumoral tracer accumulation significantly correlates with ex vivo immune cell-derived RNA signatures. • Saturable Target Specificity: *In vivo* target specificity is clinically verified via mass-dose escalation, showing complete saturation of tumor-to-plasma ratios at 604 mg.	(48)
TIM-3 (Next-Gen Co-inhibitory)	^64^Cu-NOTA-RMT3-23	Intact IgG (~150 kDa)	Preclinical Immunocompetent Melanoma Models	• Lymphocyte Tracking: Non-invasively maps the unique spatio-temporal distribution of TIM-3^+^ tumor-infiltrating lymphocytes *in vivo*. • Therapeutic Stability: Tracer accumulation remains robust and unaffected by either single-dose or fractionated radiation therapies. • Histological Mirroring: *In vivo* immunoPET quantification is highly specific and perfectly mirrored by ex vivo immunofluorescent staining.	(51)
TIGIT (Next-Gen Co-inhibitory)	^68^Ga-GP12	D-Peptide (~2 kDa)	Phase I/Exploratory Clinical Advanced NSCLC Patients	• Same-Day Workflow: D-peptide antagonist exhibits high affinity (*K*_D_: 37.28 nM) and rapid blood clearance, enabling optimal contrast at 60 min post-injection. • Clinical Translation: Successfully visualizes primary and metastatic lesions in lung cancer patients, demonstrating safety and efficacy comparable to ^18^F-FDG PET. • Heterogeneity Mapping: Non-invasively captures prominent intra-tumoral spatial heterogeneity of TIGIT density within large tumor masses.	(54)
VISTA (Next-Gen Co-inhibitory)	^89^Zr-Df-CI-8993	Intact IgG (∼150 kDa)	Preclinical (Validated for FIH) huVISTA Knock-in & Xenograft Models	• Dose Optimization: Normal tissue saturation requires a cold antibody mass dose to optimize target-specific tumor visualization. • Specific Tumor Tracking: Demonstrates robust, target-specific tracer accumulation in both humanized knock-in and VISTA-expressing human pancreatic cancer xenografts. • Massive Hematopoietic Sink: Exhibits extreme physiological tracer retention in myeloid-rich lymphoid organs (spleen uptake up to 291.9% ID/g on day 3).	(56)
ICOS (Co-stimulatory)	^68^Ga-DOTA-ICOSpep	Peptide (∼4.4 kDa)	Preclinical Humanized A549 LUAD Models	• Same-Day Kinetics: Novel peptide scaffold (*M*_w_ : 4426.9 Da) facilitates rapid clearance, enabling high-specificity activated T-cell imaging. • Therapeutic Predictor: Successfully monitors T-cell influx and predicts anti-tumor efficacy induced by local (cGAMP) or systemic (diABZI) STING agonists. • Multi-Omics Validation: PET ROI quantifications perfectly correlate with ex vivo ICOS IHC staining (*R*^2^ = 0.936), CD3 depletion, and TME proinflammatory cytokine profiles (IFN-γ, IL-6).	(61)
4-1BB (CD137) (Co-stimulatory)	^18^F-AlF-NOTA-BCP137	Bicyclic Peptide (∼2-3 kDa)	Phase I/Pilot Clinical Immunotherapy-treated HCC Patients	• Same-Day Paradigm: Hydrophilic bicyclic peptide overrides traditional antibody kinetics, allowing rapid clearance and sharp whole-body visualization within 1 d. • Early Stratification Guide: Successfully stratifies HCC conversion therapy responders before structural changes, where positive scans (TMR>3) strongly associate with extended survival. • Pathological & Toxicity Mapping: Resection histopathology validates intense tracer accumulation in activated CD8^+^ CD137^+^ T cells, while longitudinal scanning captures early immune-related adverse events (irAEs).	(65)
B7-H3 (CD276) (Stromal & Tumor)	^68^Ga-B7H3-BCH	Affibody (~7 kDa)	Phase I Clinical 20 Patients with Malignant Tumors	• Rapid Same-Day Window: Optimized affibody architecture achieves fast clearance and optimal target-to-background contrast at 50–60 min post-injection. • Surpassing FDG Efficacy: Outperforms 18F-FDG in diagnostic accuracy, resolving false negatives in differentiated HCC and metastatic gastric peritoneal lesions. • Quantitative Threshold: Tumor uptake strictly correlates with baseline cellular expression (*P*<0.005), yielding 100% specificity for high-density screening via an SUV_max_ cutoff of 3.85.	(69–74)

### 2.1 First-generation targets: the PD-1/PD-L1 axis and CTLA-4

PD-1 is natively expressed on activated and exhausted CD8^+^ T cells infiltrating the tumor microenvironment (13). Its principal ligand, PD-L1, is dynamically inducible on neoplastic cells, tumor-associated macrophages, and stromal elements under regulatory or inflammatory conditions (28). This profound spatial heterogeneity across distinct metastatic niches is precisely what standard single-site tissue biopsies systematically fail to capture (29). Consequently, target-specific ImmunoPET offers a whole-body, non-invasive, and quantitative companion alternative.

Tracer development originally converged on the direct radiolabeling of therapeutic monoclonal antibodies. Preclinical characterization validated that vectors such as ^89^Zr-pembrolizumab and ^89^Zr-nivolumab exhibit specific accumulation in target-positive lymphocytic infiltrates within the tumor parenchyma, regional lymph nodes, and secondary lymphoid structures (30, 31). The first clinical translation concurrently established the preliminary proof-of-concept in advanced entities, detailed further in Section 4.1. The subsequent decade of molecular imaging has moved along pivotal axes that early single-centre studies could not address: validating the imaging approach in larger, prospectively designed multicentre cohorts, and extending it to clinical applications beyond simple response prediction alone. The prospective multi-center Phase 0/1 trial exemplifies the first trajectory: explicitly designed to establish whether ^89^Zr-DFO-durvalumab PET/CT can characterize PD-L1 distribution at a scale suitable for larger multi-center immunotherapy networks, it has reported robust validation metrics ahead of full clinical rollout (32, 33). Furthermore, prospective expansion trials leveraging macrocyclic peptides like ^18^F-BMS-986229 during combination immunotherapy have established definitive baseline stratification thresholds, where elevated standard uptake values accurately segregate clinical responders from progressors while highlighting key discordances with concurrent single-site histopathology (34).

The pharmacokinetic constraint of intact IgGs—which require extended blood clearance windows—subsequently motivated the development of engineered fragments and smaller molecular scaffolds to maximize target-to-background contrast (23). Adnectin-based tracers like ^18^F-BMS-986192 enable robust tracking within one hour post-injection, representing a clinically mature small-scaffold paradigm (35). This structural engineering logic has since matured from preclinical concepts into validated first-in-human translation (36, 37). Prospective evaluations of camelid-derived variable domains, such as [^68^Ga]Ga-NOTA-RW102 and its albumin-binder derivatives, have demonstrated same-day visualization of differential PD-L1 topographies in thoracic oncology (38). Crucially, these single-domain platforms have unlocked previously unfeasible clinical applications, including the early prediction of severe immune-related adverse events during active checkpoint blockade. Each scaffold class carries distinct trade-offs between sensitivity, contrast, and imaging latency, a multi-dimensional consideration addressed in detail in Section 3.

CTLA-4 occupies a distinct functional position. It is constitutively expressed on regulatory T cells and transiently upregulated on activated effector T cells, primarily at the priming phase in lymph nodes. Imaging CTLA-4 thus reflects a different biological compartment than PD-1/PD-L1. Higashikawa et al. first demonstrated CTLA-4-targeted PET using ^64^Cu-DOTA-anti-CTLA-4 in CT26 tumor-bearing mice, showing significant tracer accumulation in CTLA-4-expressing T-cell infiltrates (39). Ehlerding et al. subsequently extended this to NSCLC models using ^64^Cu-labeled ipilimumab and its F(ab’)_2_ fragment, confirming that the intact antibody achieved higher absolute tumor uptake while the fragment provided superior target-to-background ratios at earlier time points (40–42). These experiments also identified CTLA-4 expression on certain NSCLC cell lines independent of T-cell infiltration—an interpretive consideration of direct relevance to clinical imaging.

### 2.2 Next-generation co-inhibitory receptors: LAG-3, TIM-3, TIGIT, and VISTA

Resistance to PD-1/PD-L1 blockade is frequently mediated by compensatory upregulation of alternative co-inhibitory pathways (43, 44). ImmunoPET targeting of these receptors addresses the need to map this resistance landscape non-invasively and longitudinally. The four targets discussed below differ substantially in expression breadth, mechanism, and clinical maturity, and are treated in turn below.

LAG-3 is a CD4 homologue broadly expressed across activated T cells, regulatory T cells, NK cells, and plasmacytoid dendritic cells, and suppresses T-cell function through dual engagement of MHC class II and fibrinogen-like protein 1 (FGL1) (45)—a multi-lineage distribution and dual-ligand mechanism that already distinguishes it functionally from TIM-3, discussed below. Its clinical relevance is no longer hypothetical: FDA approval of relatlimab plus nivolumab in metastatic melanoma in 2022 established LAG-3 as a validated combination-therapy target, creating direct translational demand for a companion imaging biomarker (46). Tracer development has progressed from single-domain antibody feasibility studies—Lecocq et al.’s ^99m^Tc-labeled nanobody showed specific splenic and nodal uptake that was absent in LAG-3-depleted mice (47)—through an intact-antibody Phase I trial (^89^Zr-REGN3767, NCT03780725) confirming biodistribution and tumor uptake in NSCLC and head and neck squamous cell carcinoma (48), to a maturing peptide-based platform that has now reached first-in-human testing. A ^68^Ga-labeled cyclic peptide tracer, [^68^Ga]Ga-CC09-1, achieved a tumor-to-muscle ratio of 17.18 ± 3.20 in preclinical models and, in a bench-to-bedside study, was translated into melanoma and NSCLC patients, where it resolved intratumoral LAG-3 heterogeneity not visible on concurrent [¹^8^F]FDG-PET (49). A separate study using the related tracer [^68^Ga]Ga-NOTA-C25 demonstrated that LAG-3 PET could detect a STING-agonist-driven “cold-to-hot” immunophenotype transition before any measurable change in tumor size—directly realizing the longitudinal resistance-mapping rationale introduced above (50).

TIM-3 differs from LAG-3 in both breadth and biological meaning: rather than marking a broad population of activated or regulatory lymphocytes, it identifies a narrower, terminally exhausted CD8^+^ T-cell state whose co-expression with PD-1—rather than PD-1 positivity alone—predicts resistance to single-agent PD-1 blockade. This distinction gives TIM-3 imaging a more specific rationale: it is not a general activation marker but a candidate biomarker for selecting patients who require combination rather than monotherapy. Wei et al.’s ^64^Cu-NOTA-RMT3-23 antibody tracer established proof of concept in syngeneic B16F10 melanoma, showing tumor uptake concordant with TIM-3 expression on TILs (51). Probe engineering has since moved toward protease-resistant peptide chemistry, paralleling a broader design trend also seen in TIGIT imaging below: a D-configured peptide tracer, [^68^Ga]Ga-DOTA-D-P24, achieved 1.6-fold higher tumor uptake than its L-configured counterpart and visualized interleukin-15-induced TIM-3 upregulation, demonstrating sensitivity to cytokine-driven target modulation in addition to baseline detection (52). Clinical translation nonetheless remains earlier-stage than for LAG-3 or TIGIT.

TIGIT is expressed on CD8^+^ cytotoxic T cells, CD4^+^ helper T cells, FOXP3^+^ regulatory T cells, and NK cells, and its suppressive function operates partly through competition with the co-stimulatory receptor CD226 for the shared ligands CD155 and CD112. Shaffer et al. compared ^64^Cu- and ^89^Zr-labeled anti-mouse TIGIT antibodies and found that ^89^Zr-TIGITmAb provided superior image contrast, with quantitative TIGIT imaging on TILs confirmed in B16 melanoma allografts (53). The most clinically advanced next-generation checkpoint probe to date followed from a different scaffold class entirely: Wang et al.’s ^68^Ga-GP12, a D-peptide TIGIT antagonist, detected primary and metastatic lesions in a first-in-human NSCLC PET/CT study with an uptake pattern comparable to [¹^8^F]FDG, while additionally resolving intratumoral TIGIT heterogeneity inaccessible to biopsy (54).

VISTA is predominantly expressed on myeloid cells within the tumor microenvironment and is enriched in immunologically cold tumors with limited T-cell infiltration (55), positioning it as a biomarker of myeloid-dominated primary resistance to T-cell-directed therapies rather than a marker of lymphocyte activation (56). Unlike LAG-3, TIM-3, and TIGIT, VISTA imaging has only recently moved past target-validation antibody tracers to dedicated, purpose-built probes, with two independent chemotypes reported within the past two years. A cyclic peptide tracer series culminating in [^68^Ga]Ga-Pip-AP1049 showed tumor uptake that correlated with the proportion of VISTA-positive cells across four syngeneic models (r = 0.8712, *P* < 0.0001) (57), while an independently developed small-molecule probe, [^68^Ga]Ga-SMIC-2501, showed comparable IHC concordance (r > 0.75) using an unrelated chemical scaffold (58). The convergence of two structurally distinct probe classes on the same quantitative relationship strengthens confidence that VISTA expression is now reliably PET-quantifiable, even though both remain preclinical and dosimetry, biodistribution in non-myeloid-rich organs, and first-in-human translation are still required before VISTA imaging can inform myeloid-targeted combination strategies in cold tumors.

### 2.3 Co-stimulatory and stromal targets: ICOS, 4-1BB, and B7-H3

Co-stimulatory receptors are upregulated upon antigen recognition and T-cell activation, making them pharmacodynamic readouts of immune engagement rather than markers of suppression—a distinction that shapes the imaging rationale for each target below (59). ICOS is substantially upregulated on CD4^+^ T cells following CTLA-4 blockade and was identified as a principal activation signature in CD19-directed CAR-T cell therapy, motivating ICOS-directed ImmunoPET as a non-invasive strategy for confirming productive co-stimulation rather than merely quantifying receptor density (60). This rationale has been validated using two complementary scaffolds developed independently: a peptide-based tracer, [^68^Ga]Ga-DOTA-ICOSpep, whose tumor uptake correlated linearly with ICOS immunohistochemistry and tracked the proinflammatory response to a STING agonist in a lung adenocarcinoma model (61); and a first-in-class anti-ICOS nanobody, [^68^Ga]Ga-NOTA-ICOS-53 (*K*_D_ = 85.3 pM), which showed concordant PET and biodistribution quantification in ICOS-expressing xenografts (62). Separately, an anti-CD8 minibody has been used to characterize the kinetics of CD8^+^ T-cell infiltration following ICOS agonist therapy alone and in combination with PD-1 blockade, showing tracer accumulation in tumor-draining lymph nodes as early as day 4—preceding intratumoral signal and illustrating how lineage-marker imaging (Section 6.2) can complement direct ICOS-targeted tracers in monitoring co-stimulatory therapy (63).

4-1BB (CD137), a TNF receptor superfamily member, is rapidly induced on CD8^+^ T cells and NK cells upon antigen encounter, offering a potential early window into productive immune priming following checkpoint blockade or combination immunotherapy (64). This rationale has progressed beyond preclinical proof of concept: a bicyclic peptide probe, [¹^8^F]AlF-NOTA-BCP137, was first validated across mouse models with varying CD137 expression and then evaluated in a pilot clinical study in patients with hepatocellular carcinoma receiving immunotherapy or combination immunotherapy, where tracer uptake predicted early therapeutic response ahead of conventional imaging—among the first direct clinical evidence that a co-stimulatory ImmunoPET probe can function as an early response biomarker, though validation in larger cohorts remains necessary (65).

OX40 (CD134), another TNF receptor superfamily costimulatory molecule, is transiently upregulated on activated CD4^+^ and CD8^+^ effector T cells within hours of antigen engagement—kinetics faster than those of 4-1BB and well suited to early pharmacodynamic readouts. The principle was established using ^64^Cu-DOTA-anti-OX40 in a CpG-oligodeoxynucleotide vaccination model, where tumor tracer uptake measured on day 2 predicted tumor regression on day 9 with greater accuracy than anatomical or blood-based measures (66). ^89^Zr-DFO-OX40 imaging subsequently extended this principle to glioblastoma vaccination models, localizing T-cell activation to the tumor-draining lymph node (11.4%ID/cc) and spleen (16.7%ID/cc) (67). Clinical translation has progressed furthest through ^89^Zr-ivuxolimab, built on a therapeutic OX40 agonist already in clinical evaluation; the tracer showed specific binding to activated human T cells and detected antigen-driven T-cell activation in a humanized OX40 mouse model (68)—positioning OX40 alongside 4-1BB as an early, increasingly clinically translatable window into productive immune priming.

B7-H3 (CD276) differs from the preceding targets in two important respects. First, it is overexpressed across a broad spectrum of solid tumors—including glioma, neuroblastoma, breast, prostate, and colorectal cancer—while maintaining limited expression in most normal adult tissues (69). Second, its expression on both tumor cells and tumor-associated vasculature makes it a target amenable to both diagnostic imaging and radioimmunotherapy (70). Burvenich et al. developed ^89^Zr-DS-5573a for B7-H3-targeted PET and demonstrated specific tumor uptake with quantification of inter- and intra-tumoral expression heterogeneity in preclinical models (71). Clinical translation has advanced furthest in neuro-oncology. Luther et al. demonstrated that ¹²^4^I-labeled omburtamab (8H9) delivered by convection-enhanced delivery in patients with diffuse intrinsic pontine glioma enabled direct PET-based dosimetry in the brain, with a subsequent Phase I trial confirming high intralesional doses with negligible systemic exposure (72, 73). This diagnostic/therapeutic pairing with ¹³¹I-omburtamab represents a clinically implemented theranostic application of B7-H3 ImmunoPET, discussed further in Section 5.2.

## 3 Probe engineering: the molecular toolbox for ImmunoPET

The clinical translation of ImmunoPET depends not only on target selection but equally on the engineering of the probe itself. Scaffold architecture governs pharmacokinetic behavior (75, 76). Radionuclide choice determines whether the imaging window is hours or days. Bioconjugation chemistry controls the quality and reproducibility of the final product (77). These three variables are interdependent: a mismatch between any of them limits image quality and clinical utility, regardless of target biology.

### 3.1 Targeting scaffold diversity: from intact antibodies to nanobodies

Intact monoclonal antibodies (~150 kDa) remain the most commonly used targeting vectors for ImmunoPET, largely because the infrastructure for their production and clinical testing is already established. Their high affinity and bivalency yield favorable absolute tumor uptake (76). The trade-off is pharmacokinetic: blood clearance is slow, driven by FcRn-mediated recycling, and optimal tumor-to-background contrast requires 5–7 days between injection and imaging (75). This mandates pairing with long-lived radionuclides such as ^89^Zr. An additional complication is Fc-mediated biological activity (78). For checkpoint-targeting antibodies used as imaging probes, immune effector function is generally undesirable. This has been addressed through isotype selection (IgG4 over IgG1) or strategic point mutations in the Fc region; the LALAPG triple mutation, for example, abrogates FcγR binding and has been shown to reduce hepatic background accumulation in ^89^Zr-labeled antibodies while improving tumor-to-liver ratios in preclinical models (79).

Engineered antibody fragments address the pharmacokinetic limitations of intact IgGs at the cost of reduced avidity and, in some cases, increased renal retention. The minibody (scFv-CH3 homodimer, ~80 kDa) and the cysteine-modified diabody (~55 kDa) have emerged as formats that balance rapid clearance with retained tumor uptake (80–82). Both are bivalent and clear primarily through the liver, yielding high-contrast images within 4–24 hours and permitting use of ^64^Cu (t½ = 12.7 h). Smaller formats—single-chain variable fragments (scFv, ~25 kDa) and single-domain antibodies (VHH/nanobodies, ~15 kDa)—clear predominantly through the kidney within 1–2 hours, enabling same-day imaging with ^68^Ga or ¹^8^F. However, high renal retention can obscure pelvic lesions and complicates dosimetry (76). Strategies to reduce renal uptake include co-infusion of gelofusine, removal of polyhistidine tags, and PEGylation, each with partial efficacy (83, 84).

Non-antibody protein scaffolds expand the toolkit further. Affibodies (~6 kDa) are among the most studied: the PD-L1-binding affibody Z~PD-L1~ radiolabeled with ¹^8^F allowed tumor imaging within 90 minutes post-injection in preclinical models (85, 86). Adnectins (~10–12 kDa), derived from the fibronectin type III domain, have advanced furthest toward clinical application among small scaffolds: ¹^8^F-BMS-986192 and its ^68^Ga counterpart are now in clinical trials for PD-L1 imaging in NSCLC and melanoma (37, 87, 88). The central determinant in scaffold selection is therefore not molecular weight per se, but rather the optimal pharmacokinetic window for a given target’s expression kinetics—and its match with an available radionuclide of appropriate half-life.

### 3.2 Radionuclide selection and site-specific bioconjugation

Radionuclide selection follows directly from scaffold pharmacokinetics. The guiding principle is simple: the physical half-life of the isotope should approximate the biological half-life of the probe. Intact antibodies require ^89^Zr (t½ = 78.4 h) or ¹²^4^I (t½ = 100.2 h). Minibodies and diabodies are well matched with ^64^Cu (t½ = 12.7 h). Nanobodies, affibodies, and peptides pair with ^68^Ga (t½ = 67.7 min) or ¹^8^F (t½ = 109.8 min). The practical consequence is that ^68^Ga and ¹^8^F are generator- or cyclotron-derived isotopes available at most nuclear medicine sites, facilitating same-day clinical workflows, while ^89^Zr requires a dedicated proton cyclotron and radiochemistry infrastructure (89–91).

^89^Zr has become the dominant radionuclide for intact antibody ImmunoPET, and the chemistry of its conjugation is now well-defined. The standard approach employs the bifunctional chelator desferrioxamine B functionalized with a para-isothiocyanatobenzyl group (DFO-pPhe-NCS), developed by Vosjan et al., which couples to lysine residues on the antibody under mild alkaline conditions (92). This random conjugation method is robust and widely adopted, but it generates heterogeneous immunoconjugates with variable chelator-to-antibody ratios and potential affinity impairment if conjugation sites overlap with the antigen-binding region. A recognized problem with DFO-chelated ^89^Zr is *in vivo* transmetallation: released free ^89^Zr deposits in bone, producing spurious skeletal signal. Next-generation chelators—DFO* and its cyclic derivative DFOcyclo*, both bearing an additional hydroxamate moiety—have demonstrated substantially reduced bone uptake compared to DFO-modified antibodies in head-to-head imaging comparisons using ^89^Zr-trastuzumab (93–95).

For ^64^Cu, macrocyclic chelators NOTA and DOTA are widely used. ^64^Cu also undergoes β^-^ emission, giving it intrinsic theranostic potential when paired with ^67^Cu (90, 96, 97). A persistent issue is transchelation of ^64^Cu to plasma proteins *in vivo*, leading to hepatic accumulation; cross-bridged macrocyclic chelators such as CB-TE2A offer improved kinetic inertness and are under active evaluation.

Random conjugation chemistry has a fundamental limitation: the heterogeneous distribution of chelators across lysine residues—dozens per antibody—yields a statistical mixture of immunoconjugates with different numbers and positions of chelators per molecule. This variability translates directly into batch-to-batch inconsistency (98). Site-specific conjugation strategies solve this by directing chelator attachment to defined, pre-engineered positions. Three approaches have achieved documented improvements. First, cysteine engineering introduces a free thiol at a defined location (typically the hinge region) for maleimide-based conjugation (77). Second, enzymatic methods using Sortase A (SrtA) recognize a short C-terminal LPXTG motif and ligate glycine-modified chelators with high regioselectivity; a study applying this to an EGFR-targeting antibody fragment demonstrated simultaneous ^89^Zr and ^64^Cu labeling at controlled sites (99). Third, a chemoenzymatic strategy combining glycan remodeling and strain-promoted azide-alkyne click chemistry (SPAAC) installs chelators specifically on the heavy chain Fc glycans, preserving the Fab region entirely. A direct comparison showed that ^89^Zr-DFO-trastuzumab produced by this chemoenzymatic route exhibited measurably higher immunoreactivity and improved tumor uptake relative to the randomly conjugated counterpart (100). Site-specific conjugation is increasingly considered the standard for GMP-grade probe production, though the added synthesis complexity remains a translational barrier (101).

### 3.3 Pretargeting strategies via bioorthogonal chemistry

Conventional direct-labeled ImmunoPET carries an inherent tension. Intact antibodies need long-lived radionuclides because blood clearance is slow—but long-lived radionuclides deliver higher radiation doses to normal tissues during the waiting period (102, 103). Pretargeting resolves this by separating the two functions: the antibody is injected first and allowed to localize over several days; only then is a small radiolabeled effector molecule injected, which finds and reacts with the pre-positioned antibody in situ. The circulating effector clears within minutes to hours, dramatically reducing background radiation (104–106). Crucially, while this paradigm holds significant clinical translation potential for resolving tracer-saturation dilemmas, pretargeted checkpoint imaging currently remains predominantly preclinical, with optimization of *in vivo* stability ongoing.

The most chemically versatile platform for *in vivo* pretargeting is the inverse electron demand Diels-Alder (IEDDA) reaction between a tetrazine (Tz) tag on the effector and a trans-cyclooctene (TCO) handle on the antibody, which operates via ultra-fast, copper-free click kinetics with rate constants exceeding 10^4^ M^-^¹s^-^¹ under physiological conditions (107). In contrast, the strain-promoted azide-alkyne cycloaddition (SPAAC) platform leverages macrocyclic cyclooctynes like dibenzocyclooctyne (DBCO) to react with organic azides. While SPAAC avoids potential copper catalyst interference during radiometal coordination, it typically proceeds with significantly slower reaction rates than the IEDDA system. Zeglis et al. demonstrated the IEDDA approach for pretargeted PET in colorectal cancer, achieving tumor uptake of 4.1 ± 0.3%ID/g at just 4 hours post-radioligand injection while substantially reducing normal tissue dose compared to directly labeled antibody (108–112).

The checkpoint imaging context gives pretargeting particular practical value. Radiolabeled therapeutic antibodies such as pembrolizumab or nivolumab carry a risk of occupying and saturating PD-1 binding sites, potentially confounding downstream immunotherapy dosing or triggering immune activation. Pretargeting separates the pharmacological and imaging functions: the non-radiolabeled antibody localizes normally, and the effector radioligand provides the PET signal without saturating additional binding sites (113, 114). This advantage becomes clinically meaningful as combination checkpoint imaging and treatment monitoring protocols enter trials. The technical challenge—ensuring sufficient TCO stability *in vivo* and optimizing the timing between antibody and radioligand injection—remains an area of active investigation, with several groups exploring modular linker designs and PK-adjusted dosing intervals (115).

## 4 Clinical translation: a tumor-type perspective

The clinical evidence base for ImmunoPET is uneven across histologies. Thoracic oncology has generated the largest and most mature dataset, with several completed Phase I/II trials now reporting predictive correlates (116, 117). Genitourinary and gastrointestinal cancers are catching up through targeted CAIX, HER2, and PD-L1 imaging programs (118–120). Hematological malignancies have benefited from the particularly favorable pharmacokinetics of radiolabeled anti-CD20 and anti-CD38 agents in the vascular compartment (121). Neuro-oncology remains the most challenging context, where the blood–brain barrier selectively excludes the large scaffolds that dominate the current clinical portfolio (122–124).

### 4.1 Thoracic oncology and melanoma

The landmark study by Niemeijer et al. enrolled 13 advanced NSCLC patients prior to nivolumab and simultaneously evaluated two tracers in the same cohort (125). ^89^Zr-nivolumab, imaged at 5–7 days post-injection, correlated with PD-1-positive TIL density by IHC; responding tumors showed higher SUVpeak than non-responding ones (median 6.4 vs. 3.9, *p* = 0.019). In a parallel acquisition at just 1 hour post-injection, the same patients were imaged with ¹^8^F-BMS-986192, an adnectin targeting tumor-cell PD-L1. Responders again demonstrated significantly higher uptake (SUVpeak 6.5 vs. 3.2, *p* = 0.03), and this PET-based association with outcome was present even in lesions where PD-L1 IHC was negative or low—directly illustrating the whole-body, heterogeneity-resolving advantage of functional imaging over single-biopsy histology, and highlighting the pharmacokinetic trade-off between slow-clearing full antibodies and fast-clearing small scaffolds in checkpoint ImmunoPET (125).

Kok et al. extended the ^89^Zr-pembrolizumab program to 18 patients with metastatic melanoma or NSCLC in a multi-centre study published in 2022. Tracer uptake was associated with objective response (p = 0.014), progression-free survival (p = 0.0025), and overall survival (p = 0.026). However, two biopsies taken from lesions with very high PET uptake (SUVmax 17.0 and 21.0) were negative for PD-1 by IHC (116). This discordance, also observed in other series, underscores a recurring interpretive challenge: high tracer uptake may reflect infiltration by PD-1-expressing immune cells rather than tumor-intrinsic expression, and rare instances of non-specific (126, 127).

^89^Zr accumulation in PD-1-negative lesions cannot be excluded entirely. ^89^Zr-atezolizumab was evaluated by Bensch et al. in 22 patients across bladder cancer, NSCLC, and triple-negative breast cancer (NCT02453984, NCT02478099). Tumor uptake was high but spatially heterogeneous—varying within and between lesions in the same patient. Complete responders had a 235% higher SUVmax than patients with immediate progression. Geometric mean SUVmax per patient was strongly associated with both PFS and OS, while pre-treatment PD-L1 IHC and RNA sequencing failed to predict these outcomes (117). This divergence between imaging and tissue biomarkers is the most consequential finding in the field: it suggests that whole-body PET captures spatial heterogeneity that biopsy systematically misses—a limitation directly quantified in histopathological studies, where discordance rates between matched biopsies and surgical resection specimens in NSCLC have reached 48%, with κ values as low as 0.218 (128, 129).

^89^Zr-durvalumab has been evaluated in patients with NSCLC and head and neck squamous cell carcinoma (HNSCC) eligible for ICI therapy. Smit et al. demonstrated tracer specificity in NSCLC by performing paired scans with and without a therapeutic predose of 750 mg unlabeled durvalumab: the scan without predose detected significantly more tumor lesions, confirming *in vivo* target engagement (130). Verhoeff et al. subsequently extended this approach to 33 patients with recurrent or metastatic HNSCC (PINCH trial, NCT03829007), confirming feasible and heterogeneous tumor uptake, though tracer uptake did not predict treatment response in this cohort (131). The clinical problem of pseudoprogression—occurring in approximately 5.0% of NSCLC and 6.4% of melanoma patients receiving PD-1/PD-L1 inhibitors per a pooled meta-analysis (16)—provides a concrete use case for ImmunoPET, given that conventional CT and ¹^8^F-FDG cannot reliably distinguish inflammatory flare from true progression. He et al. demonstrated longitudinal monitoring utility using ^89^Zr-DFO-KN035 (an anti-PD-L1 domain antibody) in 12 patients with PD-L1-positive solid malignancies: SUVmax across multiple tumor foci decreased significantly after anti-tumor treatment, providing a pharmacodynamic readout not available from anatomical imaging (132).

### 4.2 Genitourinary and gastrointestinal malignancies

Renal cell carcinoma (RCC) occupies a particular position in ImmunoPET development because its primary tumor biomarker, carbonic anhydrase IX (CAIX), is expressed with high specificity on clear-cell histology and was one of the earliest targets for radiolabeled antibody imaging. A Phase I study of ^89^Zr-girentuximab in 10 patients with suspected ccRCC demonstrated a favorable safety profile—no grade ≥3 treatment-related adverse events—and confirmed successful differentiation of ccRCC from non-ccRCC lesions in all patients, providing the dosimetric foundation for clinical scale-up (133). More recently, Verhoeff et al. showed in metastatic clear-cell RCC that combined ^89^Zr-DFO-girentuximab and ¹^8^F-FDG PET/CT at baseline predicted watchful waiting duration, identifying patients unlikely to require immediate systemic therapy (134). The culmination of this trajectory is the Phase III ZIRCON trial, in which ^89^Zr-girentuximab achieved a sensitivity of 86% and specificity of 87% for ccRCC detection across 300 patients with indeterminate renal masses, representing the first large-scale Phase III validation of ImmunoPET as a non-invasive tissue characterization tool (119). To expand this oncology pipeline, recent trials have integrated checkpoint-specific targets within this histology; a prospective cohort study confirmed that tracking whole-body macrocyclic target deployment provides a reliable framework for dynamic immunotherapy patient selection. For checkpoint imaging specifically, a Phase II trial (NCT04006522) is evaluating ^89^Zr-atezolizumab in locally advanced or metastatic RCC, with results awaited.

Gastric and gastroesophageal cancers present a distinct imaging challenge: high inter- and intra-tumoral heterogeneity of HER2 and PD-L1 expression, combined with frequent hepatic and peritoneal metastases that produce complex background signal. Lumish et al. conducted a pilot study of ^89^Zr-trastuzumab PET in 33 patients with HER2-positive metastatic esophagogastric cancer and demonstrated that PET detected lesions—including brain metastases—not identified by conventional imaging, while revealing HER2 expression heterogeneity across sites that would directly inform treatment selection (135). Parallel interest exists in PD-L1 imaging in this histology: Cytryn et al. reported a prospective pilot study of ¹^8^F-BMS-986229, an ¹^8^F-labeled next-generation macrocyclic peptide adnectin, in 10 patients with gastroesophageal cancer, finding same-day PD-L1 PET to be safe and feasible with 88% concordance with pathologic PD-L1 CPS assessment (136, 137). The core clinical question in gastric cancer—whether PD-L1-positive tumors defined by IHC adequately reflect therapeutic target engagement—remains unresolved and is precisely what whole-body checkpoint PET is positioned to address.

### 4.3 Hematological malignancies

Hematological malignancies offer a technically favorable context for ImmunoPET: the target cells circulate or localize in accessible lymphoid compartments, and the vascular pharmacokinetics of radiolabeled antibodies are in many ways better suited to imaging tumors distributed throughout marrow and lymph nodes than to deeply buried solid tumors.

The CD20/rituximab theranostic axis established the clinical proof of principle. Perk et al. demonstrated that ^89^Zr-ibritumomab tiuxetan PET/CT delineated malignant lesions in patients with non-Hodgkin’s lymphoma, with clear uptake in all previously identified tumor sites (138). Rizvi et al. showed in seven patients with relapsed B-cell NHL that ^89^Zr-ibritumomab tiuxetan scouting scans accurately predicted the biodistribution and dose-limiting organs of subsequent ^90^Y-ibritumomab tiuxetan radioimmunotherapy (139)—establishing the theranostic paradigm that continues to drive this field. Muylle et al. refined the protocol, showing that imaging without a preload of unlabeled rituximab yielded higher tumor uptake and clearer visualization in previously rituximab-treated patients with B-cell depletion, because the cold preload competes for CD20 binding sites (140). More recently, Sonanini et al. reported CD19-directed ImmunoPET in four patients with B-cell lymphoma (follicular, DLBCL, and mantle zone) using a ^64^Cu-labeled anti-CD19 monoclonal antibody, demonstrating specific *in vivo* targeting with intra- and interindividual uptake heterogeneity that correlated with IHC CD19 expression—establishing a complementary imaging approach for cases where CD20 is lost following rituximab treatment (141). In multiple myeloma, CD38-targeted ImmunoPET with ^89^Zr-daratumumab has now progressed to a Phase II trial (NCT04814615; iMMunoPET study), in which 60 patients have been accrued and preliminary data demonstrate that CD38 PET/CT identified more disease sites than ¹^8^F-FDG in 27% of participants, including cases where FDG-PET was entirely negative, highlighting the complementarity of target-specific and metabolic imaging in marrow-distributed malignancies (121).

Multiple myeloma (MM) has become one of the most actively developed contexts for ImmunoPET, driven by the clinical success of CD38-targeting daratumumab and the need to non-invasively monitor disseminated marrow disease. After extensive preclinical validation, Ulaner et al. synthesized ^89^Zr-DFO-daratumumab with radiochemical purity exceeding 99% and reported the first-in-human imaging of CD38-positive MM, demonstrating successful lesion detection at clinical sites (121). Krishnan et al. enrolled 12 daratumumab-naïve MM patients in a Phase I trial using ^64^Cu-daratumumab, confirming safety and feasibility and showing that the tracer accurately detected and excluded MM lesions (142). Two considerations complicate CD38-targeted imaging in patients already receiving daratumumab therapeutically: the therapeutic antibody saturates CD38 binding sites and may interfere with tracer uptake. This has driven interest in CD38-specific single-domain antibodies (sdAbs). Wang et al. synthesized the ^68^Ga-labeled nanobody [^68^Ga]Ga-NOTA-Nb1053, which identified all subcutaneous and orthotopic MM lesions with radiochemical yield >50% and immunoreactivity >95%; however, as the probe shares overlapping CD38 epitopes with daratumumab, its applicability in patients on active daratumumab therapy remains limited (143). CXCR4 (CD184) adds another dimension to MM imaging: ^68^Ga-Pentixafor, a cyclic pentapeptide CXCR4 antagonist, was first characterized dosimetrically in MM patients (144) and subsequently demonstrated superior sensitivity to ¹^8^F-FDG in initial clinical series (145). In comparative studies, ^68^Ga-Pentixafor was superior or equivalent to ¹^8^F-FDG in detecting lesions in up to 63% of cases (146), with a prospective cohort in newly diagnosed MM showing an overall positive rate of 93.3% versus 53.3% for FDG (*p*=0.0005) (147), additionally reflecting homing receptor status relevant to marrow trafficking and resistance.

### 4.4 Neuro-oncology and the blood–brain barrier

The intact BBB restricts molecules above ~500 Da; full-length monoclonal antibodies (~150 kDa) are effectively excluded from normal brain parenchyma. This constraint has direct clinical consequences: in the same NSCLC cohort imaged with both ^89^Zr-nivolumab and ¹^8^F-BMS-986192, brain metastases showed substantially lower tracer uptake than matched extracerebral lesions, providing direct evidence of the barrier effect (87, 125). In GBM and other CNS malignancies, immunotherapy responses can diverge substantially from systemic disease, yet intracranial biopsy for serial monitoring is prohibitively risky.

Two strategies have emerged. The first exploits tumor-induced BBB disruption. We developed [¹^8^F]AlF-NOTA-PCP2, a ¹^8^F-labeled cyclic peptide (*K*_d_ = 0.24 nM), and evaluated it in orthotopic GBM models. Serial PET after radiotherapy captured PD-L1 upregulation with uptake rising from 9.51 ± 0.73% to 12.04 ± 1.43% ID/cc after 15 Gy, and further to 13.9 ± 1.54% ID/cc after fractionated 5 Gy × 3—changes corroborated by IHC (124, 148). Compared to the earlier tracer [¹^8^F]AlF-NOTA-WL12 (PCP1), PCP2 showed superior tumor-to-muscle ratios (25.67 ± 4.12 vs. 9.11 ± 1.13, *p* < 0.001) and approximately half the effective radiation dose (0.0384 vs. 0.0713 mSv/MBq); we further demonstrated that PET-quantified PD-L1 upregulation correlated with radioresistance mediated via the PI3K–Akt–RAD51 axis (123). This brain immune-mapping paradigm has been independently validated using the macrocyclic peptide [^18^F]BMS-986229 (137, 149). First-in-human glioblastoma PET trials confirmed specific tumor-site tracer avidity that was spatially independent of contrast-enhancement on MRI, robustly demonstrating that optimized small-molecule peptides can evaluate intracranial target portfolios without depending on structural blood–brain barrier breakdown (150).

The second strategy bypasses systemic pharmacokinetics via intrathecal or convection-enhanced delivery (CED). Luther et al. first established the theranostic feasibility of ¹²^4^I-8H9 (anti-B7-H3) in DIPG (72), and Souweidane et al. subsequently reported the Phase I CED trial in 28 children, confirming that PET-based dosimetry of [¹²^4^I]-8H9 validated lesion-specific dosing >1,200-fold above whole-body exposure with no dose-limiting toxicity (73). ^89^Zr-fresolimumab, targeting immunosuppressive TGF-β in the glioma microenvironment, has also been explored clinically in recurrent high-grade glioma, with focal uptake correlating with biopsy-confirmed target expression (122). Together, these data define the state of neuro-oncology ImmunoPET: small-molecule peptide probes exploiting BBB disruption enable meaningful tumor signal and pharmacodynamic monitoring, while the B7-H3 theranostic axis—validated through CED delivery—represents the most clinically advanced paradigm for imaging-guided CNS radioimmunotherapy.

## 5 Toward clinical implementation and theranostics

The transition from research to clinical application requires solving two problems: defining quantitative PET metrics reliable enough to guide treatment decisions, and using the same antibody scaffold to deliver cytotoxic radiation once imaging confirms sufficient tumor uptake.

### 5.1 Quantitative biomarker standardization as companion diagnostics

SUV normalizes decay-corrected activity concentration to injected activity and body weight, enabling cross-patient comparison. In practice, SUV-based comparisons are confounded by scanner sensitivity and reconstruction algorithm differences, injected antibody mass dose (which directly affects receptor occupancy), lesion delineation variability, and statistical instability in low-count acquisitions. The last issue is particularly relevant for ^89^Zr-immunoPET at late time points, where noise drives systematic positive bias in SUVmax; SUVpeak—a 1 mL spherical VOI centered at maximum uptake—is now methodologically preferred for ^89^Zr studies, with newer long-axial-field-of-view (LAFOV) PET/CT systems enabling scan duration reduction of up to 50% without compromising image quality (151–153).

TBR partially corrects for inter-patient variability in tracer availability by normalizing tumor SUV to a blood pool reference (75). For ^89^Zr-immunoPET in HER2 imaging, lesion SUVmax cutoffs range from ~3 to ≥10 depending on site and preconditioning protocol; for PSMA theranostics, TBR ≥ 2 has been used as a pragmatic threshold in dose-escalation series, but absolute cutoffs require independent validation before cross-center application (120, 154).

More fundamentally, no universally accepted threshold exists for classifying a lesion as target-positive or target-negative for any checkpoint-targeted immunoPET probe. The studies reviewed in Section 4 demonstrated statistical correlations between SUVpeak and response, but lacked pre-specified dichotomous cutpoints with prospectively defined sensitivity and specificity. This reflects study scale: most checkpoint ImmunoPET trials enrolled 6–50 patients—sufficient for feasibility and directional correlation, but insufficient to derive validated diagnostic thresholds (75). Establishing such thresholds prospectively is a prerequisite for regulatory qualification of ImmunoPET as a companion diagnostic.

Two methodological developments may narrow this gap. The first is dynamic or multi-time-point imaging to capture receptor binding kinetics: the net influx rate K~i~ from Patlak graphical analysis is less sensitive to injected mass variation and blood clearance than static SUV, and the only published parametric immunoPET example using a clinical-grade agent—^68^Ga-ABY-025 in 16 metastatic breast cancer patients—demonstrated that K~i~ maps improved HER2 characterization in small liver metastases and correlated strongly with static SUVs (*R*² = 0.95) (155). The second is PET-based radiomics. In preclinical immunoPET, Rashidian et al. showed that homogeneous intratumoral CD8^+^ T-cell distribution predicted complete PD-1 blockade response while heterogeneous distribution predicted failure—a distinction invisible to SUVmax (156). Extending these approaches clinically, Lin et al. developed a machine-learning model combining intratumoral and peritumoral ¹^8^F-FDG PET/CT radiomics in 296 NSCLC patients receiving immunotherapy, achieving AUC 0.894/0.819 in training/testing cohorts with COMB-Radscore as an independent prognostic factor—demonstrating that heterogeneity quantification by PET radiomics can predict ICI response in humans (157). Extending these approaches to immunoPET-specific cohorts with standardized protocols and prospective endpoints is the field’s most immediate translational priority.

Beyond metrics, the SITC Biomarkers Committee has highlighted that adequate pre- and on-treatment biopsy tissue for longitudinal profiling is achievable in fewer than 42% of patients in prospective series (158). PD-L1 IHC, the current FDA-approved companion diagnostic, faces additional limitations: expression is dynamic, assay concordance across antibody clones is poor, and spatial heterogeneity plus non-PD-L1-mediated resistance explain why high IHC scores do not reliably predict response (158). ImmunoPET directly addresses the spatial heterogeneity limitation, but requires harmonized acquisition, reconstruction, and SUV-normalization procedures—including phantom calibration across scanners—before its outputs can function as reproducible companion diagnostic metrics in multicenter regulatory submissions (75).

### 5.2 The theranostic pivot: from ImmunoPET to radioimmunotherapy

The theranostic logic of ImmunoPET is straightforward: if a radiolabeled antibody accumulates in tumors at sufficient concentration, replacing the diagnostic isotope with a therapeutic one—while preserving the same construct and biodistribution—enables imaging-guided radiotherapy. Two FDA-approved precedents establish the regulatory template: ¹^77^Lu-DOTATATE (NETTER-1, HR 0.18 for progression) (159) and ¹^77^Lu-PSMA-617 (VISION, OS HR 0.62) (160), both of which pair a PET diagnostic ligand with a therapeutic counterpart for patient selection and dosimetry (161).

This regulatory precedent has been accompanied by a more practical shift. PET/SPECT-based patient selection—imaging the target before treating it—has moved from a theoretical advantage to standard clinical workflow in radioligand therapy. The expansion of commercial ¹^77^Lu-DOTATATE and ¹^77^Lu-PSMA-617 programs has built supporting infrastructure that did not previously exist at this scale: routine ¹^77^Lu supply chains, dosimetry-trained physics staff, and accredited PET/SPECT-guided treatment-planning pathways are now established at a growing number of nuclear medicine centers (159–161). For ImmunoPET, this infrastructure lowers a barrier that is operational rather than biological: a checkpoint- or immune-cell-directed theranostic pair entering clinical testing today can draw on existing radiopharmacy networks and dosimetry expertise rather than building them *de novo*, as earlier antibody-based radioimmunotherapy programs were required to do. This argues for deliberately incorporating ImmunoPET-defined uptake thresholds into IO trial design as a patient-selection and dosimetry tool, rather than treating imaging and therapy as separate development tracks—though, as discussed below, the immune-specific toxicity profile of checkpoint targets still requires a distinct risk calculus from tumor-intrinsic theranostic pairs.

The clinical success of radioligand therapy (RLT), particularly ¹^77^Lu-DOTATATE and ¹^77^Lu-PSMA-617, has established imaging-guided radionuclide therapy as a validated “see-and-treat” paradigm in precision oncology. Pre-therapeutic PET-based patient selection and lesion-level dosimetry have been shown to improve outcomes and reduce off-target toxicity, supporting the role of molecular imaging as a companion diagnostic in clinical workflows. PET/SPECT-based theranostics further enables standardized treatment planning and reproducible multicenter implementation (144, 145). Within this established framework, ImmunoPET extends theranostics from tumor-intrinsic targets to the immune microenvironment, enabling non-invasive assessment of immune checkpoint expression and whole-body immune cell distribution (21, 32, 103). This positions ImmunoPET as a potential companion diagnostic for immunotherapy stratification and treatment monitoring.

For antibody-based RIT, radionuclide choice is not interchangeable. β^-^ emitters (¹^77^Lu, ^90^Y) deliver cross-fire dose suited to bulky, heterogeneous tumors. ^90^Y-ibritumomab tiuxetan remains the only FDA-approved antibody-based RIT agent, with a Phase III randomized trial demonstrating superior ORR over rituximab (80% vs. 56%, p=0.002) in relapsed/refractory follicular NHL (162); Rizvi et al. validated the ^89^Zr/^90^Y scouting paradigm with a Pearson r=0.97 correlation between pretherapeutic dosimetry and on-therapy organ doses (139). α-emitters (²²^5^Ac, path length ~80 μm) deliver densely ionizing tracks ideal for micrometastatic disease. In the Phase I ²²^5^Ac-J591 trial (32 mCRPC patients), RP2D was 93.3 kBq/kg; ≥50% PSA decline occurred in 46.9%, and grade-4 thrombocytopenia at RP2D was 9.4%—far below the 56.3% historically observed with ¹^77^Lu-J591—illustrating a materially different marrow-toxicity profile (163). A key limitation of ²²^5^Ac is *in vivo* redistribution of four α-emitting daughter nuclides, depositing off-target dose particularly in salivary glands and kidneys (164).

Matched diagnostic/therapeutic isotope pairs (^89^Zr/¹^77^Lu, ^64^Cu/^67^Cu, ¹²^4^I/¹³¹I) formalize theranostics for RIT (165). Kang et al. demonstrated that ^89^Zr-daratumumab immunoPET accurately predicted the biodistribution of ¹^77^Lu-daratumumab in CD38-positive lymphoma models, and that ¹^77^Lu-daratumumab produced tumor growth inhibition concordant with the highest-uptake sites on PET (166).

Checkpoint targets introduce a fundamental complication absent in purely tumor-targeting RIT: PD-1, PD-L1, and CTLA-4 are expressed on tumor-infiltrating effector T cells, so ablative doses risk depleting the immune compartment that checkpoint blockade is designed to activate (167). This constraint defines the target-selection logic—co-stimulatory markers on activated effectors (ICOS, 4-1BB) or tumor-intrinsic targets with limited TIL expression (B7-H3) are better positioned for therapeutic translation. The ¹²^4^I/¹³¹I-omburtamab (anti-B7-H3) pair in DIPG, validated through CED Phase I (73), is the most clinically advanced instantiation of checkpoint-adjacent theranostics.

Practical barriers to scale-up—GMP radiolabeling standards, image-based marrow dosimetry, fragile ²²^5^Ac supply chains, and logistically demanding centralized labeling windows—are solvable but non-trivial, and their scope must be honestly acknowledged in trial design (75, 161, 164).

## 6 Challenges and future directions

The clinical translation of ImmunoPET is not impeded by a single bottleneck but by a convergence of constraints operating at different levels: physical resolution limits, biological complexity that confounds single-target readouts, pharmacokinetic trade-offs that cannot be simultaneously optimized, and a cost structure that demands demonstrable decision impact before reimbursement is viable. Progress has been real—Phase I/II trials have established proof-of-concept and generated the first survival correlations—but the distance between technically feasible and routinely actionable remains substantial, and closing it requires solutions at multiple levels in parallel.

### 6.1 Persistent technical and biological barriers

Nonspecific uptake and the antigen sink. The most consistent background limitation across checkpoint ImmunoPET studies is signal in the liver, spleen, and bone marrow—organs rich in FcγR-expressing myeloid cells and FcRn-expressing endothelial cells that bind circulating immunoglobulins through mechanisms unrelated to antigen engagement (75). For checkpoint targets with constitutive expression in secondary lymphoid organs (PD-1 on circulating T cells, CD38 on plasmacytoid dendritic cells), these organs additionally function as antigen sinks. This predose trade-off was demonstrated directly for ^89^Zr-pembrolizumab by Niemeijer et al. in NSCLC patients: a 200 mg cold predose substantially reduced splenic uptake but simultaneously reduced tumor lesion detectability from 19 to 10 lesions in the same patients, because excess unlabeled antibody competed for PD-1 binding in tumor infiltrates (168). No universal solution exists; a protein dose-finding phase is required for each new construct (125).

Engineering approaches partially address this. FcRn-silencing mutations reduce hepatic recycling and accelerate blood clearance, improving tumor-to-liver ratios; the LALAPG mutation abrogates FcγR binding, reducing macrophage-mediated reticuloendothelial uptake (169). Smaller scaffolds—diabodies, minibodies, nanobodies—avoid Fc-mediated mechanisms altogether, but incur renal cortex retention that can obscure pelvic disease and complicates dosimetry (170, 171). No current scaffold simultaneously resolves both background problems.

Partial volume effect and lesion size. For checkpoint targets expressed on infiltrating immune cells rather than bulk tumor mass, the signal of interest may originate from a cellular infiltrate occupying only a fraction of a lesion’s volume. Clinical PET spatial resolution (~4–6 mm FWHM) imposes a practical lower detection limit; lesions smaller than ~10–15 mm suffer SUV underestimation of up to 40% (172). A tumor with high-density TIL infiltration in a 5 mm lymph node may therefore appear erroneously negative. LAFOV PET/CT systems substantially improve sensitivity through increased photon count rates and enable total-body dynamic imaging, directly addressing this limitation (152, 153).

PET–biopsy discordance. A recurring finding—most notably in the ^89^Zr-pembrolizumab study by Kok et al., where two biopsies from high-uptake lesions (SUVmax 17.0 and 21.0) were IHC-negative for PD-1—is that PET and biopsy do not always agree (25). This discordance has at least three explanations: spatial sampling error in biopsy, free ^89^Zr from *in vivo* transchelation depositing non-specifically, and genuine biological heterogeneity in immune infiltration. Distinguishing between these requires paired multi-lesion biopsy and improved chelator design; the DFO*/DFOcyclo* family substantially reduces bone uptake from free ^89^Zr but requires prospective clinical validation in soft tissue (127).

Biological complexity of checkpoint expression. PD-L1 is simultaneously induced by IFN-γ in tumor cells, macrophages, and stromal cells; a PD-L1 PET signal therefore cannot distinguish tumor-cell from immune-cell expression without additional spatial or functional information. FOXP3, distinguishing regulatory from helper CD4^+^ T cells, is intracellular and entirely inaccessible to antibody probes (173). No single marker adequately captures T-cell activation or exhaustion state, pointing directly to the multiplexing imperative addressed in Section 6.2.

Production costs and clinical scalability. GMP-grade antibody radiolabeling—including eukaryotic expression, radiochemical purity and immunoreactivity testing, and short-shelf-life logistics—currently costs substantially more per scan than standard ¹^8^F-FDG PET. For ^89^Zr-intact antibodies, the 5–7 day imaging window requires a minimum 1-week workflow per patient. Nanobody and peptide platforms using ^68^Ga or ¹^8^F are cheaper and logistically simpler, but each requires independent target validation (161). This cost structure defines the reimbursement threshold a clinical indication must clear through demonstrable decision impact.

### 6.2 Emerging frontiers: multiplexing, AI-driven radiomics, and beyond oncology

Multiplexed ImmunoPET. Single-target imaging cannot resolve the balance between suppressive and activating signals across multiple cell populations in the TME—a fundamentally multi-dimensional biological question. Sequential ImmunoPET in the same patient (e.g., PD-1 at day 7, then CD8 at day 14) is already feasible with appropriately matched radionuclide pairs. True simultaneous dual-isotope PET exploiting ^89^Zr’s characteristic 909 keV prompt gamma alongside ^68^Ga’s absence of prompt gamma has been explored computationally but not yet clinically validated. Bispecific tracers offer a practical alternative: Moek et al. demonstrated ^89^Zr-AMG-211, a ~55 kDa CEA×CD3 BiTE, in 9 patients with gastrointestinal adenocarcinomas, showing specific accumulation in CD3-rich lymphoid tissues and clear, 9-fold inter-patient heterogeneity in tumor uptake—providing a dual-target readout from a single injection, albeit without the ability to separate the individual contributions of each binding arm (174). The next iteration—bispecific probes reporting on co-receptor pairs such as PD-1/TIGIT or CTLA-4/ICOS—is an active area of probe engineering.

Beyond checkpoint receptors themselves, ImmunoPET of immune-lineage markers provides a complementary, target-agnostic readout of TME cellular composition. CD8-targeted imaging has progressed furthest along this translational arc: a phase 2 trial in large B-cell lymphoma demonstrated that pre-treatment whole-body CD8^+^ T-cell uptake stratified subsequent time to progression in patients receiving CD19-directed CAR T-cell therapy (175). CD4 imaging contributes a complementary temporal dimension rather than a redundant one: in immunologically “cold” breast cancer models—the T-cell-poor, myeloid-dominated phenotype discussed in the context of VISTA in Section 2.2—CD8 and CD4 PET signals rose on distinct schedules (day 6 and day 2, respectively) in mice that eventually responded to checkpoint blockade (176), and a cross-species CD4 minibody study independently showed that combined PD-L1/LAG-3 blockade produced selectively higher CD4-compartment uptake in responding animals, linking CD4 PET directly to the LAG-3-mediated resistance pathway introduced in Section 2.2 (177). Myeloid-lineage imaging via CD11b has moved furthest toward therapeutic translation: a dual ^89^Zr/²²^5^Ac-labeled anti-CD11b antibody depleted tumor-associated myeloid cells in a glioblastoma model sufficiently to extend survival when combined with checkpoint blockade, and long-term survivors rejected tumor rechallenge—evidence of durable antitumor memory rather than transient cytoreduction (178). A recent preclinical comparison adds an important caveat to this broader approach: pairing a CD8 minibody with PD-L1 occupancy imaging during combined anti-PD-L1/BRD4-inhibitor therapy showed that CD8 infiltration alone did not predict tumor regression, whereas quantifying the unoccupied PD-L1 fraction did—a reminder that lineage density and target occupancy are distinct pharmacodynamic signals, not interchangeable ones (179). None of these lineage markers is checkpoint-specific, and a comprehensive treatment of immune-cell ImmunoPET lies outside this review’s scope; they are noted here because pairing a lineage tracer with a checkpoint tracer remains the most experimentally tractable near-term route toward the multiplexed imaging discussed above.

AI-driven radiomics. PET-based radiomics extracts quantitative features invisible to visual interpretation. Applied to ImmunoPET, it serves two functions. The first is heterogeneity quantification: Rashidian et al. showed in a preclinical anti-CD8 model that homogeneous tracer distribution predicted complete PD-1 blockade response while heterogeneous distribution predicted failure (156). The second is outcome prediction by integration across features: Park et al. developed a deep learning model predicting cytolytic activity score from ¹^8^F-FDG PET/CT in 29 LUAD patients receiving ICI, with higher minimum predicted CytAct per patient associated with prolonged PFS (HR 0.25, p=0.001) and OS (HR 0.18, p=0.004) (180). Translating this from metabolic FDG imaging to target-specific ImmunoPET—where the signal carries direct biological relevance to the therapeutic target—is a near-term priority. Rigorous implementation requires phantom-based harmonization across scanners, independent prospective multicenter validation with pre-specified endpoints, and adherence to SPIRIT-AI and TRIPOD-AI reporting standards; most published studies have not yet met these criteria (75).

Beyond oncology. The ImmunoPET infrastructure developed for cancer is directly applicable to inflammatory and autoimmune diseases. Bruijnen et al. demonstrated in 20 rheumatoid arthritis patients that baseline ^89^Zr-rituximab PET/CT uptake was associated with EULAR therapeutic response at week 24—establishing the principle that B-cell imaging can prospectively identify rituximab responders (181). The same probe has been extended to orbital inflammatory disease (182) and other inflammatory conditions. Perhaps the most unexpected application emerged from COVID-19: Omidvari et al. used a CD8-targeted ^89^Zr-Df-IAB22M2C minibody on a total-body PET scanner in convalescent patients, demonstrating CD8^+^ T-cell redistribution patterns distinct from healthy controls (183)—illustrating both the platform’s versatility and the unique whole-organ information available from total-body systems. The path from these proof-of-concept studies to clinical reimbursement in inflammatory diseases will require prospective trials with decision-analytic endpoints: a more demanding regulatory pathway than the surrogate endpoint frameworks available in oncology.

## 7 Conclusion

Immune checkpoint ImmunoPET has established itself well beyond the proof-of-concept phase, driven by three unique advantages over traditional histology and metabolic imaging: its capacity for systemic, non-invasive spatial heterogeneity mapping across all lesions, its utility for longitudinal dynamic monitoring of targets under treatment pressure, and its direct translatability into personalized theranostic pairing. As reviewed here, whole-body quantitative imaging of checkpoint expression uniquely captures spatial heterogeneity, predicts treatment response, and offers theranostic insights unattainable via tissue biopsy or ^18^F-FDG PET. While the radiochemistry and molecular scaffold toolkit is now mature enough to align probe pharmacokinetics with specific target biologies, the field’s remaining hurdles are clinical rather than technological. Standardizing validated quantitative thresholds, harmonizing imaging protocols, and executing prospective trials designed for regulatory qualification represent the critical next steps. To drive this field into routine precision oncology, the most immediate, actionable recommendation is the execution of rigorous, prospectively designed multicenter clinical trials utilizing cross-scanner phantom calibration; such efforts are a prerequisite to establish standard-of-care baseline thresholds and secure formal regulatory qualification for ImmunoPET as a companion diagnostic. Ultimately, the maturation of checkpoint ImmunoPET rests not on further scientific discovery, but on the systematic discipline to generate robust, reproducible evidence.
